# Development of Biodegradable GQDs-hMSNs for Fluorescence Imaging and Dual Cancer Treatment via Photodynamic Therapy and Drug Delivery

**DOI:** 10.3390/ijms232314931

**Published:** 2022-11-29

**Authors:** Sarah Reagen, Yingfen Wu, Di Sun, Carlos Munoz, Nuri Oncel, Colin Combs, Julia Xiaojun Zhao

**Affiliations:** 1 Department of Chemistry, University of North Dakota, Grand Forks, ND 58202, USA; 2 Department of Physics and Astrophysics, University of North Dakota, Grand Forks, ND 58202, USA; 3 Department of Biomedical Sciences, University of North Dakota, Grand Forks, ND 58202, USA

**Keywords:** nanoparticles, graphene quantum dots, hollow mesoporous silica nanoparticles, cancer treatment, photodynamic therapy, drug delivery

## Abstract

Recently, nano-based cancer therapeutics have been researched and developed, with some nanomaterials showing anticancer properties. When it comes to cancer treatment, graphene quantum dots (GQDs) contain the ability to generate ^1^O_2_, a reactive oxidative species (ROS), allowing for the synergistic imaging and photodynamic therapy (PDT) of cancer. However, due to their small particle size, GQDs struggle to remain in the target area for long periods of time in addition to being poor drug carriers. To address this limitation of GQDs, hollow mesoporous silica nanoparticles (hMSNs) have been extensively researched for drug delivery applications. This project investigates the utilization and combination of biomass-derived GQDs and Stöber silica hMSNs to make graphene quantum dots-hollow mesoporous silica nanoparticles (GQDs-hMSNs) for fluorescent imaging and dual treatment of cancer via drug delivery and photodynamic therapy (PDT). Although the addition of hMSNs made the newly synthesized nanoparticles slightly more toxic at higher concentrations, the GQDs-hMSNs displayed excellent drug delivery using fluorescein (FITC) as a mock drug, and PDT treatment by using the GQDs as a photosensitizer (PS). Additionally, the GQDs retained their fluorescence through the surface binding to hMSNs, allowing them to still be used for cell-labeling applications.

## 1. Introduction

Cancer is one of the leading causes of death around the world [1] and since its discovery researchers have tried multiple means of treatment to find a cure for the deadly disease. Current methods of cancer treatment such as chemotherapy and radiology, although common and effective, are aggressive to the host and as a result cause damage and stress to the patient under treatment. Physical symptoms of fatigue, hair loss, neutropenia, lymphedema, deep vein thrombosis, and psychological symptoms such as depression and memory loss are a short list of side effects brought on by traditional cancer treatment methods [2,3,4,5]. Therefore, several disciplines such as medicine, biology, and chemistry have begun to research alternative means of cancer treatment with less severe side effects for a better quality of life [6,7,8]. Nanoscience is one such discipline that has extensively researched the use of nanoparticles for cancer treatment [9]. Nanoparticles contain several unique properties that are being exploited in the hope of finding new treatment methods that are not as taxing on the patient.

One class of nanoparticles being explored are graphene quantum dots (GQDs), which are a subset of nanoparticles defined by their size range of 2–10 nm. These particles contain several key characteristics including biocompatibility, low toxicity, resistance to photobleaching, high photostability, and photoluminescence that make them applicable in biological applications [10,11]. One vital feature of GQDs is their fluorescence capabilities, which allows them to be extensively used for bioimaging applications [12,13,14]. Over recent years, GQDs have been studied for their use in photodynamic therapy (PDT) for cancer treatment [15]. GQDs contain the natural ability to generate reactive oxidative species (ROS) due to the carbonyl functional groups found on their surface, which create a cytotoxic mechanism in the target cancer cell, though the exact pathway is still unknown [16]. Under physiological conditions, cells continuously generate and eliminate ROS compounds and naturally control these fluctuating ROS levels [17]. However, disturbance in these pathways, such as excessive ROS levels, leads to DNA, protein, and lipid damage in addition to other interrupted cellular pathways [17,18]. ROS compounds are generated under exogeneous or endogenous stimuli. Endogenous ROS occur from mitochondrial interferences, either interrupted by superoxide production which influences NADPH oxidases or have reactions with nitric oxide that thus generates peroxynitrite [19,20]. Exogeneous ROS occur from environmental stress, whether it be pollutants or hemostasis imbalance. Some of these include excessive ultraviolet (UV) exposure or ionizing radiation, chemical damage from tobacco smoke, and pharmaceutical agents [19,20]. For effective PDT treatment there are three base requirements: a photosensitizer (PS), light, and oxygen [21]. Ideally for PDT, the cancer cells absorb the PS, in this case the GQDs, which are activated by light to form ROS compounds that terminate the harmful cells through either apoptosis, necrosis, or autophagy [22]. GQDs have proven to be viable candidates in previous experiments for PDT treatment as they generate ^1^O_2_ and contain fluorescence characteristics [23,24,25]. However, due to their small particle size, GQDs struggle to remain in the target area for long periods of time and also have only recently have been explored for their drug delivery applicability [26]. Furthermore, GQDs are not commonly synthesized from biomass-derived materials and over the past few years this synthesis method has now been more favored. This is because by using these naturally occurring molecules to synthesize GQDs via the bottom-up or top-down methods [27], “green” GQDs have shown to be more viable in cells with reduced toxicity [28]. Additionally, it has not been commonly explored if these synthetically green GQDs could also be used as effectively as their non-green counter parts for cancer treatment [29].

Additionally, hollow mesoporous silica nanoparticles (hMSNs) have many unique properties for drug encapsulating capacity, drug release behavior, and enhancing antitumor immunity [30]. hMSNs are known to be highly biocompatible and biodegradable due to pH sensitivity [31], allowing for the avoidance of accumulation in the human body and making them viable for drug delivery applications [31,32,33,34]. Features such as surface modifications [35], pore size [36], and pore volume [37] allow hMSNs to be tailored for the drug type which they are intended to deliver into the targeted cancer cell [38,39]. Due to the intrinsic properties of GQDs and hMSNs, they are two kinds of many nanoparticles that have been extensively researched for their cancer treatment abilities.

Recently, research has been conducted utilizing GQDs and hMSNs in tandem for dual cancer treatment via PDT and drug delivery in addition to cell imaging [40,41,42,43]. For the goals of this study, biomass-derived GQDs and biodegradable hMSNs will be synthesized together to make degradable graphene quantum dots-hollow mesoporous silica nanoparticles (GQDs-hMSNs) that are also uniquely efficient at drug delivery, ROS formation, and fluorescence imaging. GQDs are synthesized in one of two ways: top-down or bottom-up [27,44]. The top-down method starts by breaking down carbon material using methods such as lithography and oxidation and bottom-up combines smaller carbon-containing compounds using methods such as pyrolysis and hydrothermal heating. For this project, GQDs will be synthesized from biomass-derived material [45] by dissolving the organic compound and subjecting the solution to heat for an extended time. For hMSNs synthesis, due to the vast number of synthesis methods that all impact the hMSNs’ ability to treat cancer effectively, it remains an important and innovative field of research. This study will use the Stöber method [46] which has successfully synthesized MSNs in the past and use Na_2_CO_3_ to hollow out the MSNs [43] into hMSNs.

A variety of analytical instrumentation were used to characterize the new GQDs-hMSNs. Firstly, their size distribution was examined using transmission electron microscopy (TEM) and dynamic light scattering (DLS) due to nanoparticles being defined by their size range of one dimension being 1–100 nm [47] and previous studies showing that nanoparticle size range greatly impacts their cell interactions [48,49]. Elemental composition analysis was determined using X-ray photoelectron spectroscopy (XPS) to confirm the presence of silicon, oxygen, and carbon along with Fourier transform infrared spectroscopy (FT-IR) to confirm bond formations. Zeta-potential was determined due to solution stability and surface charge playing vital roles in cellular interactions, as charged particles typically are better ingested by cells than noncharged particles [50,51]. Then, their ultraviolet-visible (UV-Vis) absorption and fluorescence features were collected to facilitate fluorescence imaging. Their biodegradability was also tested using various pH solutions [32] to mimic biological environments. Lastly, utility was assessed for in vitro fluorescence cell imaging using confocal microscopy, ROS formation using cell viability tests for type II PDT treatment due to ^1^O_2_ formation via GQDs [52,53] and drug delivery by examining loading and releasing capabilities into the targeted cancer cells using fluorescein (FITC) dye as a mock drug [54].

## 2. Resultes and Discussion

### 2.1. Design of GQDs-hMSNs and Synthesis

The primary focus of this study is to derive nanoparticles from biomass that help support their biocompatibility and biodegradation. Using *cis*-3,4-di(furan-2-yl)cyclobutene-1,2-dicarboxylic acid (CBDA-2), a biomass-derived molecule, for synthesizing the GQDs yields synthetically green and environmentally friendly graphene-based particles. Additionally, with CBDA-2 being synthesized from agricultural waste provides the means to recycle waste products into potentially vital nanoparticles for cancer imaging and treatment. Furthermore, the nanoparticles need to be biodegradable as to avoid accumulation in the patient body that could eventually lead to toxicity. Stöber method-based silica nanoparticles have previously been shown to possess excellent biocompatibility [55,56,57] and, with their pH sensitivity, are prone to biodegradation [57,58,59] in a matter of days or weeks. These key features make them valuable to avoid host accumulation and toxicity over time as the human body will naturally break down the silica for use in biological pathways.

**Scheme 1:**
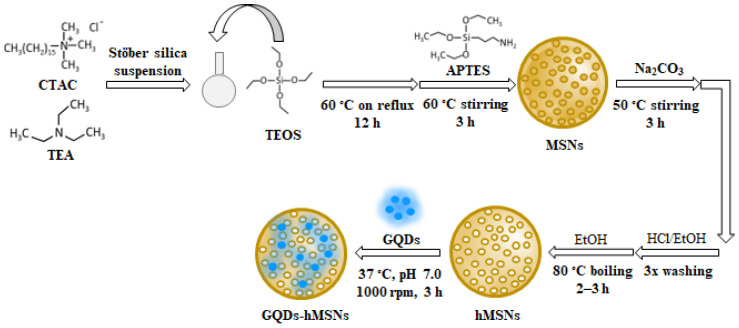
Synthesis of GQDs-hMSNs using Stöber silica suspension and previously synthesized GQDs from biomass-derived material.

To start, cetrimonium chloride (CTAC) and triethylamine (TEA) were mixed together before a Stöber silica suspension was spiked in. The solution was allowed to react before tetraethyl orthosilicate (TEOS) was added. After heating for 12 h on reflux at 60 °C, (3-aminopropyl)triethoxysilane (APTES) was added to introduce amine functional groups to the particle surface to help in biocompatibility, low pH stability, and agglomeration, and the solution was allowed to react for another 3 h while stirring at 60 °C. This resulted in mesoporous silica nanoparticles (MSNs). Na_2_CO_3_ was added, and the solution was stirred for another 3 h at 50 °C before being washed with a solution of hydrochloric acid/ethanol (HCl/EtOH) three times. EtOH was added to resuspend the particles and allowed to boil for 2–3 h at 80 °C. These steps etched out the silica core of the MSNs to make hMSNs. Once the EtOH evaporated, the hMSNs were dried to a solid. 1.0 mg was dissolved in 1.0 mL of deionized-water (DI-water) and 0.2 mg GQDs were added before the solution was diluted to 5.0 mL total volume with 20 mM 2-[4-(2-hydroxyethyl)piperazin-1-yl]ethanesulfonic acid (HEPES) pH 7.0 buffer. The solution was vortexed for 3 h at 1000 rpm before being centrifuged and dried to purify the final product of GQDs-hMSNs (Scheme 1).

### 2.2. Characterization of GQDs-hMSNs

#### 2.2.1. Size Distribution and Surface Morphology

**Figure 1:**
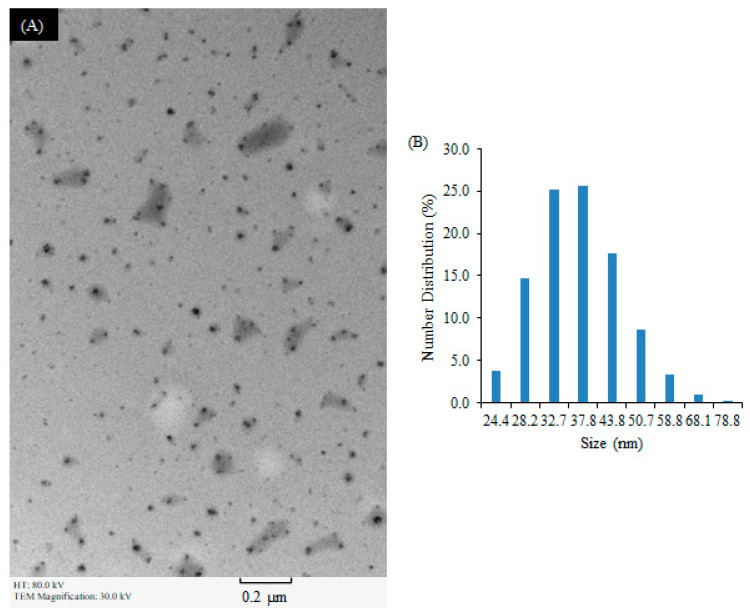
(**A**) TEM image of 80 µg/mL GQDs-hMSNs. (**B**) DLS of 32 µg/mL GQDs-hMSNs in DI-water after 5 min sonication.

Particle size is essential to identify for nanoparticles as it is the determining factor as to whether or not they can be classified as nanoparticles. It is also important to determine particle size for biological applications as larger nanoparticles can struggle to diffuse across the cell membrane for imaging or drug delivery. Smaller particles tend to diffuse quicker and easier across the cell membrane; however, they are more common to cause cell toxicity [60]. Therefore, two different analyses were conducted to determine the size distribution of the GQDs-hMSNs. TEM images showed particles ranging from 20–50 nm (Figure 1A). The larger particles were more difficult to differentiate between single particles and smaller clusters. Thus, DLS was also used to determine particle size, averaging 47.0 ± 19.0 nm (Figure 1B).

**Figure 2:**
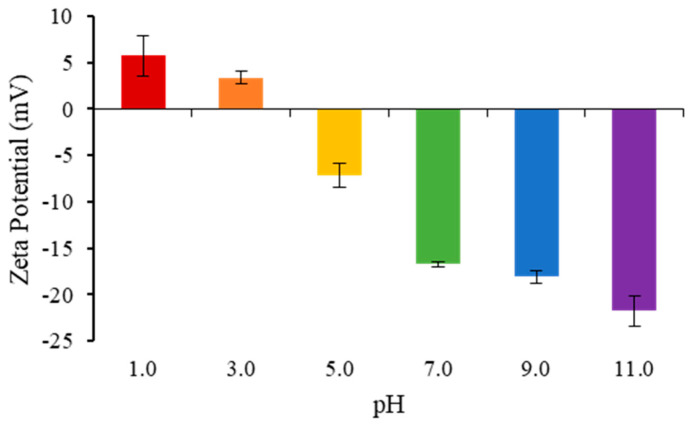
Zeta potential of GQDs-hMSNs (32 µg/mL) in various pHs (maleate 1.0, citrate 3.0–5.0, HEPES 7.0, CHES 9.0, and CAPS 11.0).

Surface charge is also essential for nanoparticle determination as the charge will impact cellular uptake. Since uncharged particles are more difficult for cells to ingest, it is important to ensure and analyze the surface charge of the GQDs-hMSNs. Therefore, zeta potential measurements were taken at various pHs (1.0–11.0) and depict a decreasing potential from +5.71 to −21.8 mV (Figure 2). The most agglomeration and solution instability occurred in the more acidic pHs of maleate and citrate (1.0–5.0) but increased in stability from pH 7.0–11.0 (HEPES, 2-(cyclohexylamino)ethanesulfonic acid (CHES), and 3-(cyclohexylamine)-1-propanesulfonic acid (CAPS), indicating an overall negative surface charge. Lower agglomeration and greater solution stability in neutral pHs benefit biological applications. Additionally, higher agglomeration was observed for prolonged sonication times, greater than 30 min, and at concentrations higher than 50 μg/mL.

#### 2.2.2. Functional Group Formation and Elemental Composition

**Figure 3:**
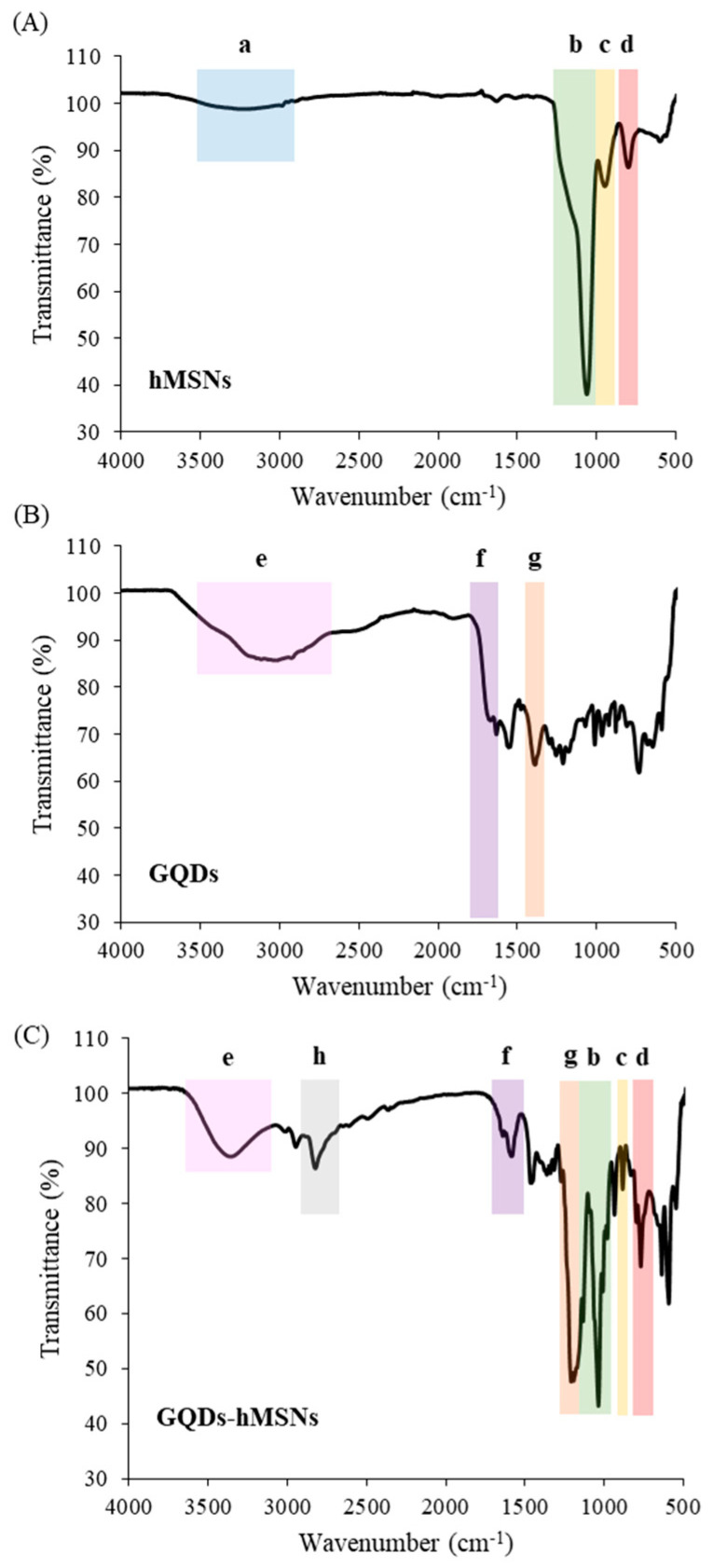
FT-IR spectra of hMSNs (**A**), GQDs (**B**), and GQDs-hMSNs (**C**). Peaks are (**a**, blue) Si-O-H stretching, (**b**, green) Si-O-Si bending, (**c**, yellow) Si-OH vibration, (**d**, red) Si-O vibration, (**e**, pink) -OH, (**f**, purple) C=O, (**g**, orange) C-O/C-N, and (**h**, gray) C-H.

Functional groups are essential for biological applications and additionally provide insight into the surface composition of the newly synthesized GQDs-hMSNs. FT-IR spectra were collected for hMSNs, GQDs, and GQDs-hMSNs to compare the differences of functional groups and additional bond formation after the synthesis of the nanoparticles and quantum dots together (Figure 3). For hMSNs without additional GQDs, functional groups were determined to be Si-O-H stretching (3222 cm^−1^, Figure 3A(a)), Si-O-Si bending (1058 cm^−1^, Figure 3A(b)), Si-OH vibration (944 cm^−1^, Figure 3A(c)), and Si-O vibration (796 cm^−1^, Figure 3A(d)) [61,62,63]. GQDs functional groups have been identified as -OH (3114 cm^−1^, Figure 3B(e)), C=O (1668 cm^−1^, Figure 3B(f)), and C-O/C-N (1384 cm^−1^, Figure 3B (g)). The spectrum of GQDs-hMSNs contained both bonds found in hMSNs and GQDs, indicating the compilation of the two nanoparticle species, along with an additional C-H bond formation (2821 cm^−1^, Figure 3C(h)). Furthermore, the -OH and C-O peaks are more distinct than what is observed in the GQDs spectrum, along with a decrease in C=O. This indicates the breaking of C=O bonds and the formation of Si-O-C bonds and potential C−Si bonds between the GQDs and hMSNs; which signifies that the GQDs are chemically bonding to the surface of the hMSNs rather than being absorbed into the pores [64,65]. This is beneficial as the GQDs are less likely to escape the hMSNs before being delivered into the cancer cells for imaging and PDT treatment in addition to not interfering or interacting with the mock loading drug for delivery.

**Figure 4:**
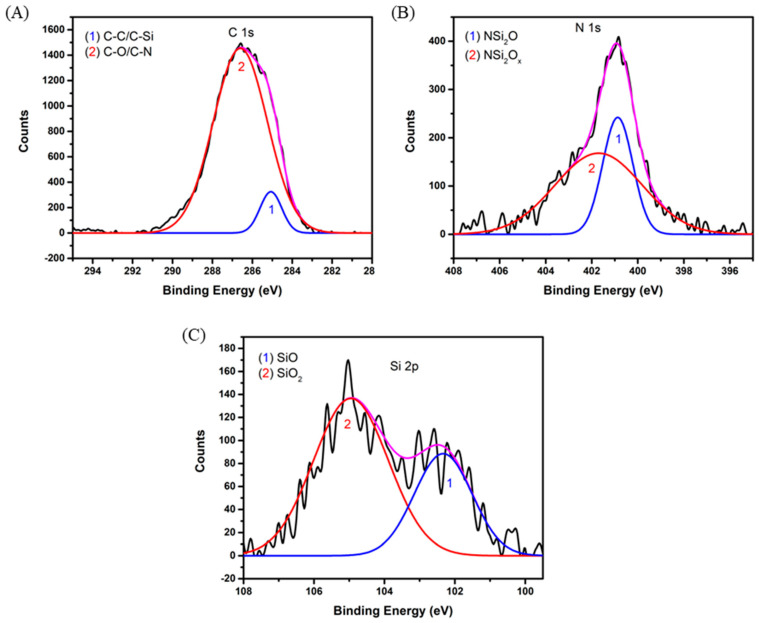
XPS high-resolution scans of (**A**) carbon, (**B**) nitrogen, and (**C**) silicon peaks in GQDs-hMSNs. The carbon peak shows C-O, C-Si, C-C and C-N bonds. The nitrogen peak is primarily NSi_2_O_x_. Lastly, the silicon peak shows SiO_x_ bonds.

XPS analysis was also conducted to confirm the elemental composition and bond formation of the GQDs-hMSNs seen in the FT-IR spectra and ensure that silicon, oxygen, and carbon were all present in the new synthesized particles. These three primary elements were identified in addition to the presence of nitrogen. This nitrogen content was attributed to APTES bonded to the hMSNs surface [66]. The elements of silicon and oxygen were attributed to be from the hMSNs, carbon and oxygen from the GQDs, and nitrogen from APTES. High-resolution scans of the carbon, nitrogen, and silicon peaks of the GQDs-hMSNs showed C-O/C-N and C-C/C-Si bonds within the carbon peak (Figure 4A), NSi_2_O and NSi_2_O_x_ in the nitrogen peak (Figure 4B), and the silicon showed SiO and SiO_x_ bonds (Figure 4C). The presence of the C-Si bond supported the spectrum seen in FT-IR and confirmed the chemical binding of GQDs to the hMSNs surface, and further explained the slight peak shift in the excitation wavelength.

#### 2.2.3. Optical Properties and pH Effects

**Figure 5:**
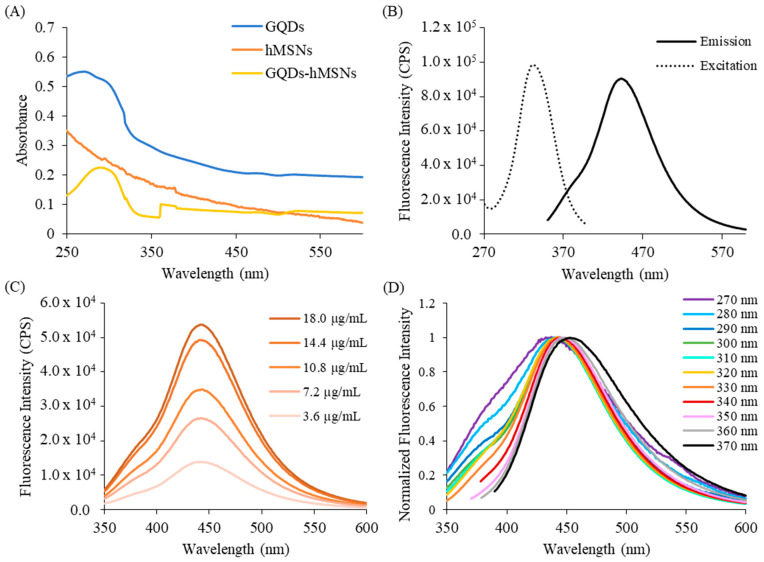
(**A**) Absorption spectra of GQDs (100 µg/mL), hMSNs (10 µg/mL), and GQDs-hMSNs (10.8 µg/mL). (**B**) Excitation and emission spectrum of GQDs-hMSNs (10.8 µg/mL). (**C**) Concentration dependency of GQDs-hMSNs. (**D**) Changing excitation of GQDs-hMSNs (18 µg/mL).

Cell labeling for confocal microscopy imaging require fluorescent characteristics of nanoparticles in order to be applicable. Thus, the absorption, excitation and emission spectra were collected of the GQDs-hMSNs. Previously, the GQDs contained an absorbance of 300 nm which was attributed to the C-C bonds π-π* transition as well as the *n*-π* of the oxygen-containing groups [67]. However, after conversion with hMSNs, the new GQDs-hMSNs spectral features slightly shifted, with an absorbance at 310 nm (Figure 5A) and an excitation peak at 330 nm (Figure 5B) due to chemical binding on the silica surface. The emission peak at 440 nm remained the same (Figure 5B). Additionally, the GQDs-hMSNs showed concentration dependence (Figure 5C) as the GQDs have shown previously. However, the radiation dependency and red-shifting of the emission peak with increasing excitation wavelength (270–370 nm) did not occur as drastically as analyzed before (Figure 5D), and thus the GQDs now displayed a better rapid internal conversion from higher initial excited states to lower states that prevented the red-shift since being attached to hMSNs.

**Figure 6:**
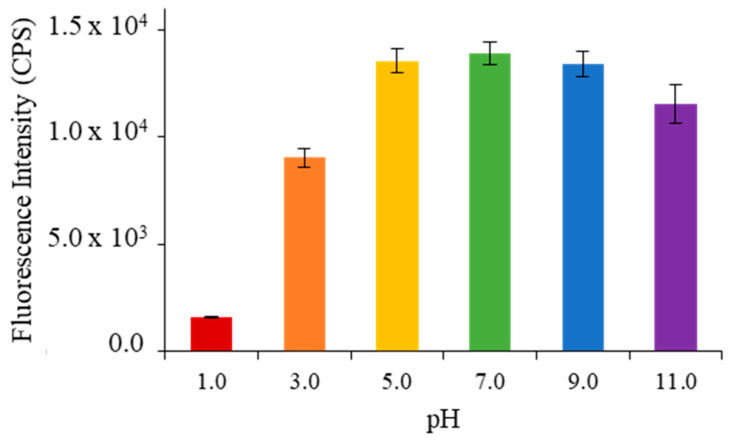
GQDs-hMSNs with changing pH (maleate pH 1.0, citrate 3.0–5.0, HEPES 7.0, CHES 9.0, CAPS 11.0) of the GQDs-hMSNs (36 µg/mL). The same trend was observed for GQDs, where quantum dots quench at pH 1.0 but remain steady in the neutral pHs.

The human body contains a range of pHs, therefore the GQDs-hMSNs are required to show fluorescence stability across different pHs and avoid quenching to optimize in vitro cell imaging. Therefore, the GQDs-hMSNs fluorescence was analyzed at pHs 1.0–11.0 for any quenching tendencies in addition to test their stability and agglomeration characteristics in this wide range of pH. Due to the negative charge of the GQDs-hMSNs as seen in Section 2.2.1, more acidic environments could potentially cause the particles to destabilize and lose their surface charge. When this happens to particles, they will agglomerate and fall out of suspension. Additionally, they will lose their fluorescent signature and forfeit their use in cell labeling applications. For these GQDs-hMSNs, at pHs 5.0–9.0, the nanoparticles retained their fluorescence and were only slightly quenched at pH 11.0. Furthermore, pHs 1.0 and 3.0 showed greater quenching, with pH 1.0 nearly losing fluorescence altogether (Figure 6). However, this is typical of GQDs as extreme acidic conditions lead to agglomeration and solution instability, as previously mentioned, primarily due to excessive positive charge of the solution caused the negatively charged particles to destabilize. This was also seen in the zeta potential analysis (Section 2.2.1). Nonetheless, the highest fluorescence observed in the neutral pHs was encouraging for cell labeling applications as the fluorescence will be maintained in the biological pHs.

### 2.3. GQDs-hMSNs Biodegradation

A goal of this project is to lessen immune system taxation via traditional cancer treatments and therefore it is important to ensure that the GQDs-hMSNs are not retained long-term in the patient body and are broken down after a couple of weeks; or at least after they have fulfilled their purpose for drug delivery and as a PS agent for PDT treatment. Solutions of 10 mM phosphate-buffered saline (PBS) buffer, pH 7.0 and 6.5, and 10 mM citrate buffer, pH 5.0 and 4.0, were used to analyze the biodegradation of the GQDs-hMSNs to mimic biological systems. The GQDs-hMSNs were added to the buffer pHs (0.2 mg/mL) and vortexed for 6 days at 37 °C, 1000 rpm. Samples were taken every 24 h and analyzed via DLS and TEM to observe particle degradation over time.

**Figure 7:**
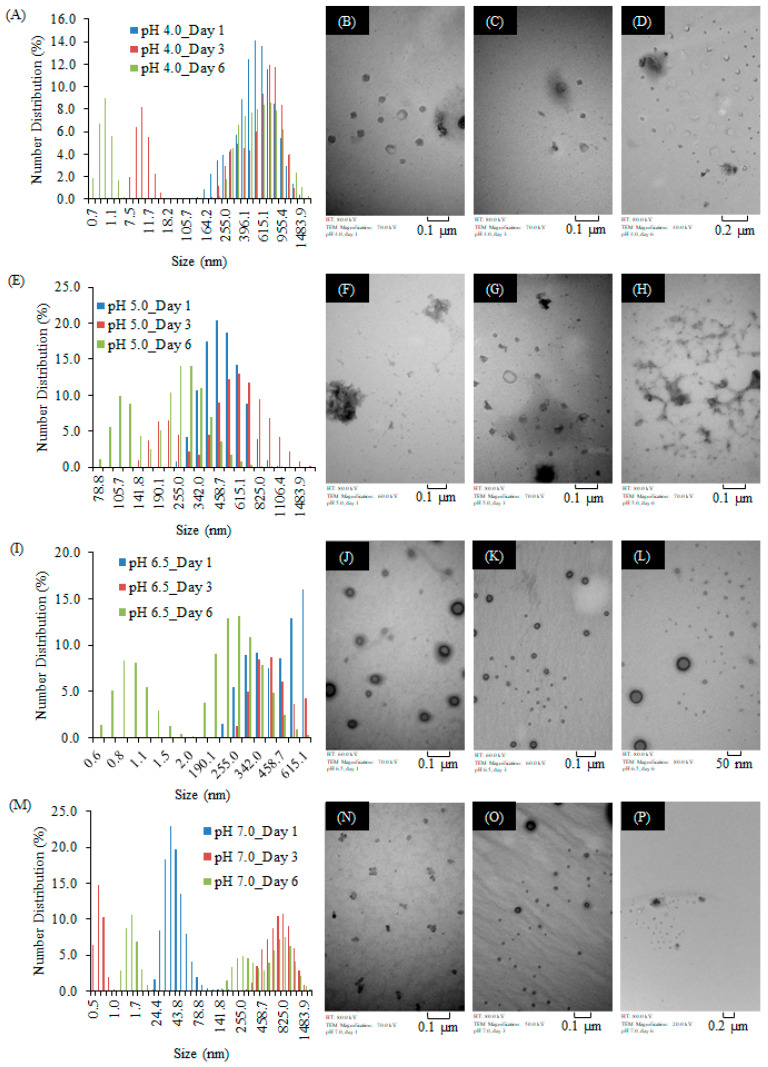
(**A**) DLS of 32 µg/mL GQDs-hMSNs particle degradation at pH 4.0 for 6 days. TEM images of particles at pH 4.0 after (**B**) day 1, (**C**) day 3, and (**D**) day 6. (**E**) DLS of 32 µg/mL GQDs-hMSNs particle degradation at pH 5.0 for 6 days. TEM images of particles at pH 5.0 after (**F**) day 1, (**G**) day 3, and (**H**) day 6. (**I**) DLS of 32 µg/mL GQDs-hMSNs particle degradation at pH 6.5 for 6 days. TEM images of particles at pH 6.5 after (**J**) day 1, (**K**) day 3, and (**L**) day 6. (**M**) DLS of 32 µg/mL GQDs-hMSNs particle degration at pH 7.0 for 6 days. TEM images of particles at pH 7.0 after (**N**) day 1, (**O**) day 3, and (**P**) day 6.

DLS of pH 4.0 revealed particle agglomeration after the first 24 h in the buffer (Figure 7A). The TEM image of day 1 depicts large, swollen particles with some already rupturing under the acidic conditions (Figure 7B). For day 3, the particles were approximately the same size as on day 1, as observed in the DLS. However, more debris particles emerged in lower quantities (Figure 7C). Lastly, day 6 TEM depicted a large increase in debris particles along with breakage and misshaped larger particles as they continued to degrade (Figure 7D). For pH 5.0, a similar trend was observed for the first day, with increased particle agglomeration observed in the DLS (Figure 7E). Under TEM, day 1 GQDs-hMSNs at pH 5.0 depicted the same enlarged particles. However, they appeared to aggregate together instead of swelling like at pH 4.0 (Figure 7F). By day 3, the particles at pH 5.0 TEM (Figure 7G) were similar to day 3 particles at pH 4.0 while the DLS also confirmed slight degradation of the particles although they were degrading more slowly. Day 6 TEM (Figure 7H) showed rupturing particles that clumped together to yield larger size distributions observed in DLS.

PBS buffers showed slightly different particle formations. For pH 6.5, the particles immediately aggregated and swelled after one day, as observed in DLS and TEM (Figure 7I,J). However, the swelling was more severe than in the more acidic buffers. Day 3 TEM images showed a small decrease in particle size (Figure 7K), with less swollen particles and the appearance of smaller degradation debris particles. Day 6 TEM (Figure 7L) showed even more debris particles, as seen in the DLS.

In comparison, pH 7.0 showed a similar trend as pH 6.5. After day 1, the DLS results (Figure 7M) showed the same particle size distribution as observed previously (Section 2.2.1). However, the TEM images for day 1 (Figure 7N) showed burst or rippled particles, indicating that even after a short period of time, the particles started to degrade and lose their surface integrity. Day 3 for pH 7.0 (Figure 7O) showed increased particle agglomeration and swelling with smaller debris particles, much like pH 6.5 on day 6. Lastly, for day 6 at pH 7.0, there was an increase in debris particles with some swollen and agglomerated particles (Figure 7P).

Therefore, the GQDs-hMSNs have a prolonged degradation at pH 7.0 compared to 4.0. Thus, they will function longer at a natural, healthy pH, allowing the particles more time to accumulate in cancerous cells without releasing the chemotherapy drug or GQDs. Furthermore, as observed in the zeta potential measurements (Section 2.2.1) the GQDs-hMSNs show agglomeration and solution instability in acidic pHs. This will contribute to the increased agglomeration seen at pH 5.0 and 4.0 after one day of exposure. From there, the particles degraded faster at pH 4.0 based on the particle size in TEM. However, retained agglomeration at pH 5.0 for day 6 yielded a larger particle size in DLS than what was observed in TEM. Furthermore, pH 7.0 and 6.5 showed less severe particle agglomeration after one day, as expected due to the zeta potential measurements previously discussed. Nonetheless, the slightly more acidic pH of 6.5 showed larger particle size and agglomeration, initially, compared to pH 7.0 and showed a faster rate of degradation. Therefore, the GQDs-hMSNs will most likely degrade faster in cancerous environments that are more acidic, releasing the chemotherapy drug and GQDs into the cancer cells for treatment and imaging.

### 2.4. Cell Viability, In Vitro Cell Imaging, PDT Treatment, and Mock Drug Delivery of GQDs-hMSNs

**Figure 8:**
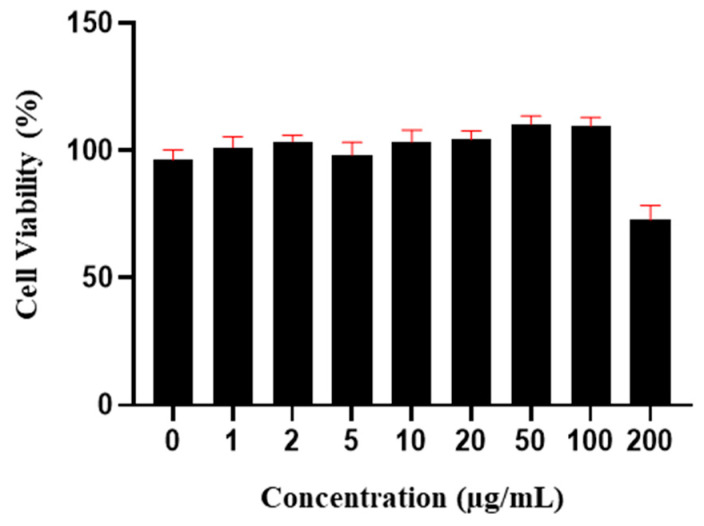
LDH assay cytotoxicity analysis of GQDs-hMSNs up to 200 μg/mL in RAW 264.7 cells.

In order to determine the biocompatibility of the GQDs-hMSNs, cytotoxicity tests were also analyzed using LDH assays. Effects of GQDs-hMSNs on cell viability were tested using RAW 264.7 cells, incubating the cells for 24 h with different concentrations of GQDs-hMSNs. Thermos Fisher CyQUANT™ LDH Cytotoxicity Assay Kit was performed to measure cell viability. The cells showed no signs of toxicity up to 100 μg/mL. However, at 200 μg/mL the cell counts decreased to 70–60% of the original amount (Figure 8). This is attributed to the hMSNs as the GQDs showed no cytotoxicity up to 200 μg/mL in previous studies [67].

**Figure 9:**
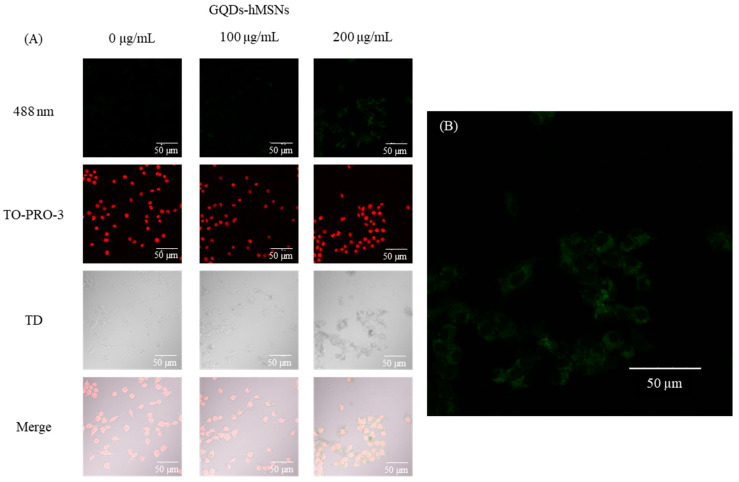
In vitro images of RAW 264.7 cells with GQDs-hMSNs. (**A**) 0, 100, and 200 μg/mL cell cultures with TO-PRO-3 labeled nucleus. (**B**) 200 μg/mL GQDs-hMSNs using the 488 nm laser showed a weak fluorescence signal. Transmitted (TD) and merged images depict the cell integration of GQDs-hMSNs in the cytoplasm.

Furthermore, to determine GQDs-hMSNs applicability in cell imaging, in vitro confocal microscopy images were collected using RAW 264.7 cells. The cells were incubated for 4 h with 100 and 200 μg/mL GQDs-hMSNs (Figure 9A). Additionally, TO-PRO-3 dye was used to label the cell nucleus. Upon imaging, weak fluorescence signal was found using the 488 nm laser for the GQDs-hMSNs (Figure 9B). This is due to the 488 nm laser being just outside the GQDs optimal excitation wavelength. However, the concentration of GQDs added in the synthesis can be increased to bind more GQDs to the surface of the hMSNs to provide a stronger fluorescent signal in the cells.

**Figure 10:**
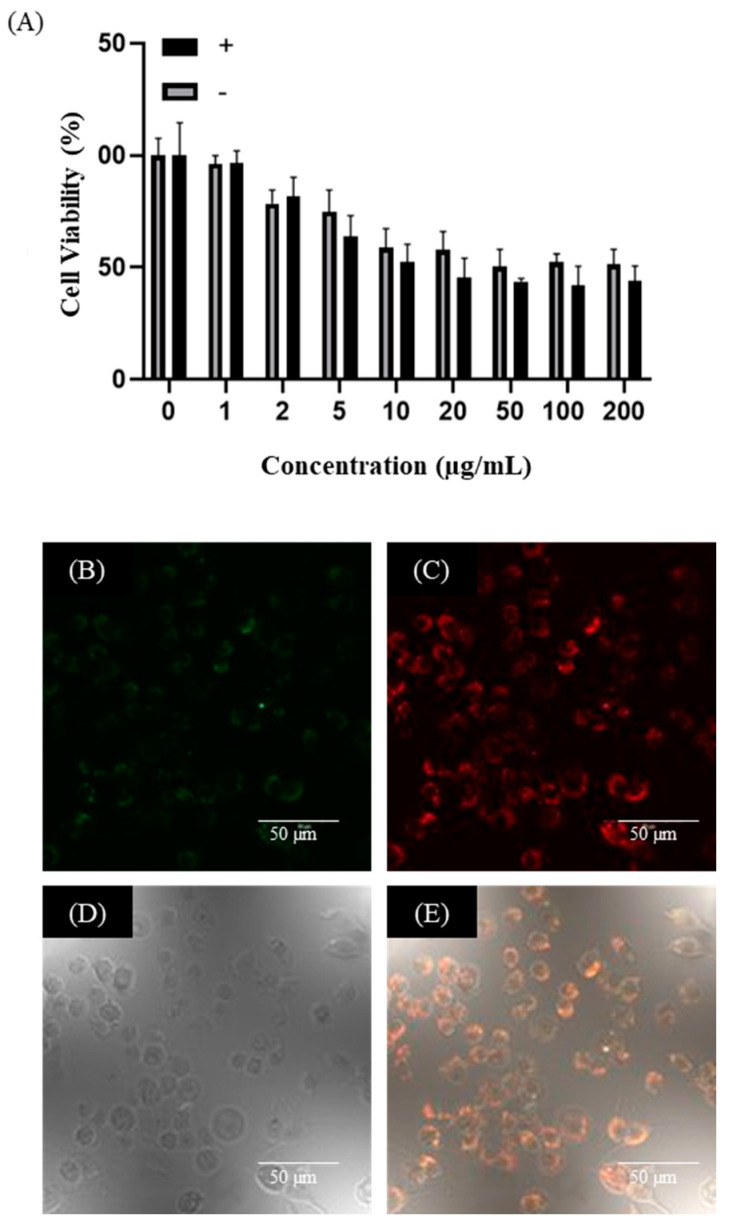
PDT treatment of RAW 264.7 cells with GQDs-hMSNs. (**A**) LDH assay cytotoxicity analysis after 24 h treatment at different concentrations (0–200 μg/mL); (+), 785 nm laser irradiation; (−), no laser irritation. Confocal microscope images of LysoTracker Red with 200 μg/mL GQDs-hMSNs (**B**) 488 nm laser, (**C**) 561 nm laser, (**D**) TD, (**E**) merged.

The GQDs-hMSNs were also examined for their use in PDT treatment of RAW 264.7 cells using LysoTracker Red in confocal microscopy and LDH assay for cytotoxicity analysis. A concentration range of 0–200 μg/mL GQDs-hMSNs was tested and after 30 min of laser irradiation at 11.81 mW/cm^2^, the cells were incubated for another 24 h. The cytotoxicity showed a linear decrease in cell viability with increasing concentrations of GQDs-hMSNs until 20 μg/mL, signifying that the GQDs-hMSNs were generating ^1^O_2_ and induced toxicity in the RAW 264.7 cells (Figure 10A). Concentrations higher than 20 μg/mL decreased cell viability compared to the control, however, the decrease was similar to the 20 μg/mL concentration. This decrease in ^1^O_2_ generation is attributed to the induced toxicity of the hMSNs observed in the cytotoxicity analysis previously discussed. The cells were subjected to a greater toxicity due to the presence of hMSNs before the GQDs could generate ^1^O_2_. Therefore, fewer viable cells would be available for PDT treatment and display a decrease in effective PDT treatment, but not necessarily due to a lack of ^1^O_2_ generation. Additionally, LysoTracker Red was used to track toxicity due to the ROS generation within the RAW 264.7 cells. Confocal microscope images were taken using the 488 nm laser (Figure 10B) for GQDs-hMSNs and the 561 nm laser (Figure 10C) for LysoTracker Red. The TD (Figure 10D) and merged (Figure 10E) images showed that the GQDs-hMSNs and LysoTracker Red are in the RAW 264.7 cells’ cytoplasm. This signified that the ROS generation came from the incubated GQDs-hMSNs, which were the main contributor to the cells’ decrease in viability.

**Figure 11:**
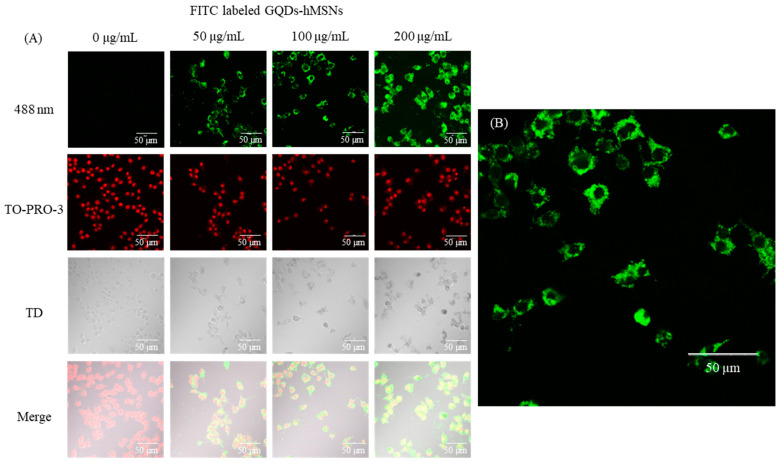
In vitro images of RAW 264.7 cells with GQDs-hMSNs doped with FITC dye as the mock drug. (**A**) 0, 50, 100, and 200 μg/mL cell cultures with TO-PRO-3 labeled nucleus. (**B**) 100 μg/mL GQDs-hMSNs with FITC.

Drug delivery via GQDs-hMSNs using FITC dye as a mock drug was also analyzed by confocal microscopy. Concentrations of 0, 50, 100, and 200 μg/mL GQDs-hMSNs@FITC were cultured with RAW 264.7 cells for 4 h. The results showed an increasing fluorescence signal with increasing concentration of GQDs-hMSNs@FITC (Figure 11A), confirming that FITC is being delivered by the GQDs-hMSNs. Lower concentrations, such as 100 μg/mL of the GQDs-hMSNs@FITC still adequately delivered the mock drug and yielded exceptional cell images (Figure 11B). Due to the increased toxicity of the GQDs-hMSNs above this concentration, lower amounts of the nanoparticles can still be effectively used as a drug carrier.

## 3. Methods and Materials

### 3.1. Materials and Sample Preparations

A Millipore water purification system (18.3 Ω cm) was used to produce DI-water. 3-(cyclohexylamine)-1-propanesulfonic acid (CAPS buffer, >99%), 2-(cyclohexylamino)ethanesulfonic acid (CHES buffer, >99%), 2-[4-(2-hydroxyethyl)piperazin-1-yl]ethanesulfonic acid (HEPES buffer, 99.5%), citric acid (>99.5%), maleic acid (>99%), phosphate-buffered saline (PBS buffer, tablet), dimethyl sulfoxide (DMSO, >99.7%), triethanolamine (TEA, >99%), tetraethyl orthosilicate (TEOS, 98%), cetyltrimethylammonium chloride (CTAC, 25 wt. % in H_2_O), (3-aminopropyl)triethoxysilane (APTES, 99%), and cyclohexane (anhydrous, 99.5%) were purchased from Sigma-Aldrich (St. Louis, MO, USA). RAW 264.7 cells cell line was purchased from ATCC (Manassas, VA, USA). Cell culture media, penicillin-Streptomycin and trypsin were purchased from Gibco (Waltham, MA, USA) 4% paraformaldehyde (PFA) was purchased from Electron Microscopy Sciences (Hatfield, PA, USA). Fetal bovine serum (FBS) was purchased from Peak Serum, Inc (Wellington, CO, USA). Fluoromount-G mounting media was purchased from Southern Biotech (Birmingham, AL, USA). 96-well plate, CyQUANTTM LDH Cytotoxicity Assay kit, Invitrogen LysoTracker TM Red DND-99, and To-Pro-3 were purchased from Thermo Fisher (Waltham, MA, USA). Cell culture plates were purchased from Greiner Bio one (Kremsmunster, Austrian). Lab-Tek II Chamber Slide system was purchased from Nalge Nunc International Corp. (Naperville, IL, USA). The Micro cover glass was purchased from Sargent-Welch VWR Scientific (Buffalo Grove, IL, USA). Olympus FV3000 Laser Scanning Confocal Microscope was purchased from Olympus Corporation (Shinjuku City, Tokyo, Japan). EXL800 microplate reader was purchased from Bio Tek (Winooski, VT, USA).

Buffers of maleic acid, citric acid, HEPES, CHES, and CAPS were prepared in 20 mM concentrations, and additional 10 mM PBS and citrate, in DI-water with the pH being adjusted by the addition of HCl or NaOH. Zeta potential and DLS samples were subjected to sonication for 5–10 min before analysis. For the drug delivery application of the GQDs-hMSNs, FITC was utilized as the mock drug. A 5.3 µM solution in DMSO was prepared for drug delivery applications. Degradation analysis was conducted in 10 mM PBS buffer, pH 6.5 and 7.0, to mimic a biological environment. The GQDs-hMSNs were added to both buffers, vortexed at 1000 rpm for 3 h at 37 °C for 6 days, and sampled on the first, third, and sixth day. Solutions were analyzed by DLS and TEM to confirm particle degradation over time.

### 3.2. Instrumentation Used for the Characterization and Analysis of GQDs-hMSNs

A Hitachi 7500 transmission electron microscope (TEM) was used to image the GQDs-hMSNs. A Malvern model of Nano-ZS Zetasizer was used to measure size distribution and the zeta potential. UV-Vis absorption was analyzed by a Perkin Elmer Lambda 1050 UV-250 UV/Vis/NIR spectrophotometer and the fluorescence spectra was acquired by a Shimadzu RF-6000 spectrophotometer. IR spectra of hMSNs, GQDs, and GQDs-hMSNs were collected on a Thermo Fisher Scientific Nicolet iS5 Fourier transform infrared spectrometer (FT-IR). A Benchmark MultiThermal (Cool—Heat—Shake) H5000-H vortex system was used to integrate of GQDs into the hMSNs. A PHI Model 10-360 electron spectrometer with non-monochromatized Al Kα (1486.3 eV) X-rays was used for X-ray photoelectron spectroscopy (XPS). An Olympus FV3000 Laser Scanning Confocal Microscope was used for the in vitro cell imaging of the GQDs-hMSNs with two channels being used for imaging: red (Fura-Red (Ca-free)) and green (Alexa 488).

### 3.3. Synthesis of GQDs-hMSNs

GQDs were synthesized as they were in previous experiments [67]. To summarize, 20 mg CBDA-2 [68] was dissolved in 20 mL DI-water with the pH adjusted to 10.0 using NH4OH to help CBDA-2 dissolve before autoclaving for 12 h at 200 °C. The resulting solution was then dialyzed for 12 h using a 100–500 Da membrane against DI-water.

hMSNs were synthesized first by mixing 24 mL of CTAC and 0.16 mL TEA into 40 mL of DI-water. CTAC and TEA were dissolved before 2.5 mL of solid silica suspension (prepared by the Stöber method) was added and transferred to a round bottom flask. Next, 20 mL of a 10 *v/v*% solution of TEOS in cyclohexane was added to the round bottom flask in an oil bath on reflux at 60 °C. After 12 h, 2.0 mL of 10 *v/v*% APTES in cyclohexane was added and the solution was stirred for another 3 h at 60 °C. Then, the heat was lowered to 50 °C and 120 mL of 0.2 M Na_2_CO_3_ was added and allowed to stir for 3 h. The flask was removed from the oil bath and the solution was centrifuged for 30 min at 12,000 rpm at 20 °C. The supernatant was discarded and the remaining solid was washed with 20 mL HCl/EtOH (1:10) solution three times to remove unreacted chemicals, particularly CTAC as it has great toxic effects to cells [69]. EtOH was added after the last washing step and placed back into a round bottom flask and heated in an oil bath until the EtOH reached a boil for 2–3 h. The final product of hMSNs was then dried completely to a solid for further use.

10 mg of hMSNs were added to 10 mL of DI-water and sonicated for 5 min to completely dissolve (1.0 mg/mL) and then filtered through a 0.1 µm syringe. Then, 1.0 mL of the filtered hMSNs was added to 1.0 mL of GQDs (0.2 mg/mL) and 3.0 mL of 20 mM HEPES buffer (pH 7.0) and mixed at 1000 rpm at 37 °C for 3 h before being dried at 60 °C to a solid.

### 3.4. Cell Toxicity

A total of 1.0 mL of 5.3 µM FITC (2.06 µg) was added to 1.0 mg GQDs-hMSNs in 4.0 mL of 20 mM HEPES buffer (pH 7.0) and vortexed at 1000 rpm for 3 h at 37 °C. The solution was then centrifuged at 10,000 rpm to separate any FITC not absorbed by the GQDs-hMSNs. Then, the particles were resuspended in 20 mM HEPES buffer pH 7.0 for absorption spectroscopy to quantify how much FITC was absorbed. Compared to the control, on average, 75% of FITC added was absorbed by the GQDs-hMSNs (GQDs-hMSNs@FITC).

Cell viability of GQDs-hMSNs was tested with CyQUANTTM LDH Cytotoxicity Assay kit. Briefly, the cells were plated into a 96-well plate overnight. A gradient concentration of GQDs-hMSNs (0, 1, 2, 5, 10, 20, 50, 100 and 200 µg/mL) was added into the wells. After 24 h incubation in a CO_2_ incubator at 37 °C, 10 µL of 10X Lysis buffer was added into 3 wells of 0 µg/mL group to serve as the maximum LDH controls. After 45 min, 50 μL aliquots of all groups were transferred to a new 96-well plate, mixed with 50 μL of the Reaction Mixture solution. After 30 min incubation at room temperature protected from light, 50 µL of Stop Solution was added to the wells, and the absorbance read using an EXL800 microplate reader to quantify cell viability.

### 3.5. Cell Imaging, PDT, and Drug Delivery

Cell imaging of GQDs-hMSNs or GQDs-hMSNs was investigated by adding 0, 100, and 200 µg/mL GQDs-hMSNs in to an 8-well slide chamber with cells inside. After a 4 h of incubation, the wells were rinsed with 1× PBS for 15 min 3 times, then fixed with 4% PFA for 15 min. After additional washes with PBS, To-Pro-3 was added to stain the nuclei for 15 min. After washing with PBS, the chamber was removed and the slide was covered with a cover slide by mounting medium. When dry, the slides were imaged using an Olympus FV3000 Laser Scanning Confocal Microscope.

PDT analysis of GQDs-hMSNs was conducted using a LysoTracker Red lysosome kit and LDH assay. For cell viability assays, cells were seeded into a 96-well plate and incubated overnight. GQDs-hMSNs were then added in concentrations of 0, 1, 2, 5, 10, 20, 50, 100 and 200 μg/mL and incubated for 4 h before being washed with 1× PBS buffer. The cells were then subjected to 785 nm laser irradiation for 30 min at 11.81 mW/cm^2^ and then incubated for another 24 h before analysis of the LDH assay kits. For confocal imaging, RAW 264.7 cells were seeded in an 8-well slide chamber and incubated overnight. After incubation with 200 µg/mL GQDs-hMSNs for 4 h, the cells were washed with 1× PBS buffer and after a 30 min incubation, fresh medium was added before for confocal imaging.

## 4. Conclusions

hMSNs were successfully synthesized using the Stöber method that allowed GQDs to be incorporated onto their surface via sonication to form 47.0 ± 19.0 nm GQDs-hMSNs for cell imaging and dual cancer treatment. The fluorescence characteristics of the GQDs-hMSNs were comparable to GQDs, showing pH sensitivity in acidic conditions, and zeta potential measurements showing particle agglomeration at pH 1.0–3.0, and concentration dependency. Particle degradation was investigated at pH 7.0, 6.5, 5.0, and 4.0 after being vortexed for 6 days at 1000 rpm, 37 °C. After 6 days, it was observed that the GQDs-hMSNs at pH 7.0 degrade at a slower rate compared to pH 4.0, indicating that the particles would degrade faster in cancer cells and would not linger in the patient to induce toxic effects.

The GQDs-hMSNs showed slight cytotoxic effects at 200 μg/mL, attributed to the hMSNs. With the fluorescence characteristics of the GQDs being maintained, the GQDs-hMSNs were successful absorbed by RAW 264.7 cells after 4 h of incubation to yield fluorescent in vitro images. Furthermore, RAW 264.7 cells were successfully imaged using the GQDs-hMSNs. However, higher concentrations of GQDs were needed as the 488 nm laser was out of range for optimal excitation of the GQDs-hMSNs. This issue can be addressed the last step of the synthesis by adding greater concentrations of GQDs to the hMSNs. Adding more GQDs to the GQDs-hMSNs will also aid in the toxicity analysis, as less GQDs-hMSNs need to be added to increase the fluorescence signal if a greater concentration of GQDs are attached. Furthermore, the hMSNs have shown great adaptability to the GQDs, and thus a higher concentration of GQDs can be added during the synthesis to increase their concentration for cell imaging without increasing the concentration of hMSNs to help reduce toxicity. PDT treatment was also successful using the GQDs-hMSNs, showing induced toxicity with increasing concentration as more ^1^O_2_ was generated. Additionally, the hMSNs also displayed excellent drug loading and release characteristics, utilizing FITC dye as the mock drug. RAW cells were incubated with the GQDs-hMSNs doped with FITC, and the resulting confocal images displayed excellent delivery of FITC into the cells.

## Data Availability

The authors confirm that the data supporting the findings of this study are available within the article.
